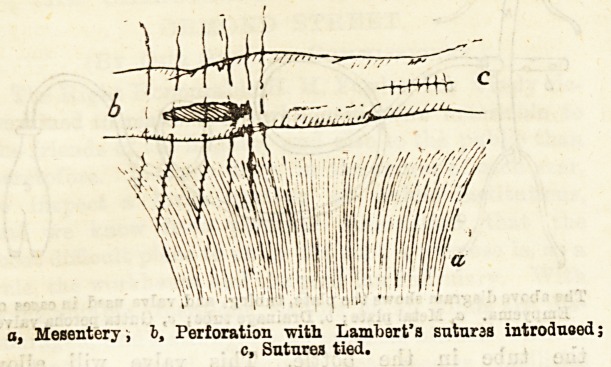# Treatment of the Bowel in Operations for Strangulated Hernia

**Published:** 1893-03-11

**Authors:** 


### ROYAL INFIRMARY, EDINBURGH.

### Treatment of the Bowel in Operations for Strangulated Hernia.

Not the least important, and often the most difficult step in the operation for strangulated hernia is to decide what is to be done with the bowel after the strangulation has been relieved by division of the constricting element.

The choice of procedures is practically between (1) returning the bowel into the abdominal cavity; (2) forming an artificial anus; (3) resecting the gangrenous knuckle of bowel; or (4) performing some plastic operation on the gut itself.

Which of these measures will be adopted depends entirely on the condition in which the bowel is found when the sac is opened—because in almost every case the sac should be opened. As no two cases of hernia are ever exactly alike, no absolute rule can be formulated which will apply universally, but the indications laid down by Sir James Paget are so clear and so sure that no apology is necessary for quoting them: "You are to judge chiefly from the colour and the tenacity. Use your eyes and your fingers; sometimes your nose; very seldom your ears, for what you may be told about time of strangulation, sensations, and the rest, is as likely to mislead you as to guide aright. As to odour, I am disposed to say that you may return intestine of any colour short of black, if its texture be good, if it feels tense, elastic, well filled out, and resilient, not collapsed or sticky; and the more the surface of the intestine shines and glistens, the more sure you may be of this rule. When a piece of intestine is thoroughly black, I believe you had better not return it, unless you can be sure that the blackness is wholly from extravasated blood. It may not yet be dead, but it is not likely to recover; and even if it should not die after being returned, there will be the great risk of its remaining unfit to propel its contents, and helping to bring on death by what appears very frequent—distension and paralysis of the canal above it. But, indeed, utter blackness of strangulated intestine commonly tells of gangrene already; and of this you may be sure if the black textures are lustreless, soft, flaccid, or viscid, sticking to the fingers, or looking villous. Intestine in this state should never be returned. . . . Then as to texture of the intestine, it should be for safety of return thin walled, firm, tense, and elastic, preserving its cylindrical form, the softer and more yielding the more pulpy, or like wet leather or soaked paper, the less is it fit for return."

Guided by these principles, the surgeon will have little difficulty in deciding how to deal with the strangulated gut:—

(1) Should examination prove it to be healthy, the knuckle is carefully sponged with an aseptic fluid, or washed with a stream of tepid boracic acid, and gently slipped back into the abdominal cavity. The next step is to close the aperture in the peritoneum and abdominal parietes, and most surgeons prefer to do so by performing one or other of the many available operations for the radical cure of the hernia.

(2) In cases where the condition of the bowel is more than doubtful, the affected portion is pulled down into the wound and left there. At this stage the surgeon has to decide whether he will open the bowel at once to permit of the escape of its contents, leaving the constricting element undivided so as to prevent faeces, &c., reaching the peritoneum; or whether he will divide the stricture, pull the bowel out and fix it in position, and wait for some hours till inflammatory adhesions shut off the peritoneal cavity before opening the damaged intestine. Of the two methods, the latter is the one usually chosen in this hospital as being the safer for the patient. To prevent peristaltic action the patient is given full doses of opium, and nothing else. A light aseptic absorbent dressing is applied for 8, 12, or 16 hours, and then the bowel is emptied by opening into it. Frequent dressing and irrigation are necessary to ensure that no septic material lodge about the wound, lest the delicate adhesions be broken down and the peritoneal cavity infected.

(3) In cases where a considerable length of bowel has been damaged beyond hope of recovery, it may be resected. The abdominal cavity is shut off by packing tightly around the extracted bowel warm sponges or pads of wool sewn into fine gauze bags. The bowel is occluded above and below the gangrenous area by means of clamps, and by an incision which includes a V-shaped portion of mesentery the unhealthy bowel is removed. All bleeding is arrested, the ends brought together by sutures, with the aid of decalcified bone plates, bobbins or tubes and the wound dressed.

(4) In many cases of strangulated hernia the bowel is perforated at or near the constricting band, a small oval aperture being formed, usually in the long axis of the gut and near the mesenteric attachment. In such cases, the method employed by Mr. F. M. Caird, of this hospital, gives excellent results. The bowel is cleansed, the edges of the aperture inverted, and the two peritoneal surfaces brought in contact by a series of Lambert's sutures. In Mr. Caird's hands this method has been most satisfactory, the risk of stricture of the bowel being so slight as to be practically ignored.

a, Mesentery; b, Perforation with Lambert's sutures introduced; c, Sutures tied.

## Figures and Tables

**Figure f1:**